# Age and gender distribution of Hepatitis C virus prevalence and genotypes of individuals of physical examination in WuHan, Central China

**DOI:** 10.1186/s40064-016-3224-z

**Published:** 2016-09-13

**Authors:** ZhiLi Niu, PingAn Zhang, YongQing Tong

**Affiliations:** Department of Laboratory Science, Renmin Hospital of WuHan University, Wuhan City, 430060 Hu Bei Province China

**Keywords:** Hepatitis C virus, Genotypes, Subtype, Prevalence, Age and gender

## Abstract

Approximately 170 million people in the world are infected with Hepatitis C virus (HCV). There are no published population based studies about the prevalence of HCV genotypes and the associations of genotype and Infection frequency with gender and age in WuHan. We aimed to investigate the distribution of HCV prevalence and genotypes among different gender and age patients with chronic HCV infection in WuHan from 2011 to 2015. A total of 2685 anti-HCV positive serum samples from individuals of physical examinationwere recruited from the Renmin Hospital of WuHan University, Hubei Province in China from January 2011 to December 2015. From these 2685 anti-HCV positive serum samples, 496 samples were with a positive PCR for HCV RNA. The number of HCV infection showed an increase with year, but the annual infection rate has remained similar (χ^2^ = 2.94, *P* = 0.568). 2685 cases were infected with HCV from 2011 to 2015 in WuHan city of China. Blood transfusion (18.14 %) was the main routs of transmission, followed by Surgery (8.94 %). The highest prevalence of HCV infection was at the age group 50–59 (25.85 % of 2685) and the lowest prevalence was 0–9 (0.93 % of 2685). HCV genotype 1 was the most prevalent (73.39 %), followed by genotypes 2 (17.14 %), 3 (5.25 %) and 6 (3.22 %). Genotype 4 and 5 was not detected in these patients. The most prevalent subtype was subtype 1b (71.98 %), followed by genotypes 2a (17.14 %). Five patients had mixed infection across the HCV subtypes. Among all genotypes, genotype 1 was highest in both male (73.27 %) and female (73.47 %) patients, followed by genotype 2. Genotype 1 (male: 29.84 % of 496, vs female: 43.55 % of 496, χ^2^ = 20.07, *P* < 0.0001), genotype 2 (male: 6.25 % of 496, vs female: 10.89 % of 496, χ^2^ = 6.81, *P* = 0.009), and 6 (male: 1.41 % of 496, vs female: 1.81 % of 496, χ^2^ = 0.626, *P* = 0.401) were more common in female patients than males, while no significant gender differences were observed for genotype 6. Among age group 50–59 years Genotype 1 was most common in male patients (29.05 % of 148) followed in 20–29 years (23.65 % of 148), genotype 2 in the age group 60–69 (12 cases of 31) and genotype 3 in the age group 50–59 (4 cases of 13) and genotype 6 was most frequent in the age group 30–39 (4 cases of 7). The frequency of HCV prevalence was significantly higher in female patients compared to males before ages 60, while the opposite result was observed after 60 years. The most common HCV genotype in WuHan was subtype 1b followed by 2a and more common among women than males patients. Further studies are needed to collect a large number of samples to estimate the different epidemiology of the HCV genotypes, because the sample size of non-genotype 1b and 2a is not large enough and other factors like disease history/monthly income/etc. are not included in our study.

## Background

Hepatitis C virus (HCV) infection is one of the most important *Flaviviridae* infections with significant clinical problems. Mohd Hanafiah et al. found that globally the prevalence and number of people with anti-HCV has increased from 2.3 to 2.8 % between 1990 and 2005 and according to recent World Health Organization data the overall prevalence of HCV infection is 0.2–2.2 % in developed countries and nearly 7 % in developing countries, with over 170 million people infected worldwide (Hajarizadeh et al. 2013; Mohd Hanafiah et al. 2013). Analysis of the HCV genome shows a remarkable genetic heterogeneity among HCV isolates from all over the world and the main risk factor is exposure to infected blood or blood products, unsterile needle-sharing among intravenous drug users (IVDU), and needle stick injuries in health care workers (Aceijas and Rhodes 2007). To date, at least six major genotypes of HCV and over 67 different subtypes on the amount of nucleotide variation (a, b, c, etc.) (Pfaender et al. 2014; Smith et al. 2014), which have different geographical distribution (Afridi et al. 2014; Madalinski et al. 2015; Marascio et al. 2014). Although HCV genotypes 1, 2 and 3 are prevalent worldwide distribution, their relative prevalence varies from one geographic area to another (Gower et al. 2014). HCV subtypes 1a and 1b are the most common in the United State of America and Europe, while subtype 1b is most commonly found in Japan (Gower et al. 2014; W. H. Organization 2016). HCV Subtypes 2a and 2b are mostly found in North America, Europe, and Japan while 2a/2c is found mainly present in Southern Italy (Cornberg et al. 2011; Germer et al. 2011; Petruzziello et al. 2013, 2014). Subtype 3a, which is very common among intravenous drug abusers, is found mainly in Europe, the USA and South East Asia (Messina et al. 2015; Zein 2000). Genotype 4 prevails in North Africa and Middle East and genotypes 5 is endemic in South Africa and Hong Kong while Genotype 6 is dominant in South China, Myanmar, Laos, Vietnam and Cambodia (Messina et al. 2015; Jang and Chung 2011; Lu et al. 2005; Nguyen et al. 2010).

Due to lack of vaccine and effective therapy, the prevention of HCV infection has been a great challenge, especially in China, which is the largest developing country and owns one-fifths of the world’s population. Presently, the prevalence rate of anti-HCV in China is reported to be 3.2 %, and about 30 million individuals are infected with HCV (Tanaka et al. 2011). At present, 1b and 2a are the major HCV subtypes circulating in China, especially in the North and West, and genotype 6 is common in Hong Kong (Chan et al. 2011; Ju et al. 2015; Wang et al. 2016; Yang et al. 2016). With global travel increasing and the population migrating, the geographic distribution has been changing in China. For example, genotype 6a has replaced 2a to become the second genotype in Guangdong because of socio-economic advancement and migration flow (Rong et al. 2014).

WuHan, the capital of Hubei Province, is located in the central China and serves as the political, economic and cultural center of the province. As one of the country’s key scientific and educational bases, WuHan enjoys the third biggest scientific and educational capabilities among major Chinese cities. It is home to 52 universities including WuHan University and Huazhong University of Science and Technology with an enrolment of 0.7 million university students. To data, there is little information available in literature about HCV genotype distribution in WuHan to date, especially in the epidemiology and HCV genotype distribution of different age and gender. Hence, the aim of this study was to provide information on the frequency of HCV infection and genotypes and the correlation with different age and gender in WuHan from 2011 to 2015.

## Methods

### Participants and samples

A total of 2685 anti-HCV positive serum samples from individuals of physical examination were recruited from the Renmin Hospital of WuHan University, Hubei Province in China from January 2011 to December 2015. From these 2685 anti-HCV positive serum samples, 496 samples with a positive PCR for HCV RNA. The age of anti-HCV positive individuals of physical examination ranged from 1 to 90 years old. All tested positive for anti-HCV by Abbott Architect Anti-HCV assay (Abbott, Chicago, USA). Inclusion criteria was positive results for anti-HCV. The exclusion criteria was the healthy people or HCV infected patients in our study without positive serum HBV antibody and HBV-DNA. Through the questionnaire survey to collect participants basic information, such as route of infection, history of Surgery, underlying disease, addiction to drugs or previous abuse and so on. Written informed consent was obtained from all participants before the collection of biological samples, and the protocol was approved by the Medical Ethics Review Committee of Renmin Hospital of WuHan University.

### Samples collection and HCV RNA extraction

Five milliliters of venous blood was collected from patients into EDTA-containing vacutainer tubes and plasma were separated by centrifugation and stored at −80 °C until tested for HCV genotyping. HCV RNA was extracted from 200 μL of plasma using QIAamp Viral RNA Mini Kit (Qiagen, Germany), according to the manufacturer’s instructions.

### RT-PCR

The cDNA was synthesized from viral RNA in a 20 μL reaction volume including 5 μL RNA and 1 μL Random primer at 65 °C for 6 min, then 4 μL Reaction Buffer, 2 μL dNTP Mixture, 1 μL RNase inhibitor, 1 μL Reverse transcriptase, 6 μL RNase Free H_2_O (Takara, Dalian, China) at 42 °C for 60 min, followed by 72 °C for 7 s. HCV fragments were amplified using a PrimeSTAR Kit (Takara, Dalian, China) in a 20 μL reaction volume including cDNA 2 μL, primer 1 μL, mix 10 μL, 7 μL ddH_2_O was performed with 1 cycle 42 °C for 5 min, 95 °C 3 min, followed by 40 cycles, each consisting of 94 °C 30 s, 56 °C 50 s, 72 °C 1 min, then 72 °C 10 min, 4 °C 10 min. All manufacturer protocols were followed. Linear range is 1 × 10^3^–5 × 10^7^ IU/mL, The lowest sensitivity is 5 × 10^2^ IU/mL.

### HCV genotyping analysis

The PCR products of partial Core and NS5B were purified (ExpinGel SV, GeneAll Biotechnology, Seoul, Korea) and sequenced using an Applied Biosystems (ABI) PRISM Big Dye Terminator Cycle Sequencing Ready Reaction Kit Version 3.1, the same PCR primers, and an ABI 3500 DX Genetic Analyzer (Applied Biosystems, Foster City, CA, USA). The sequences were aligned with reference sequences retrieved from GenBank Database using ClustalX v.2.1 (Larkin et al. 2007). HCV genotype reference sequences were retrieved from the HCV database (http://hcv.lanl.gov/content/sequence/HCV/ToolsOutline.html). The following sequences in GenBank were used as references in the phylogenetic analysis: M62321 and M67463(1a), D90208 and M58335(1b), D00944 and AB047639(2a), AB031663(2k), D17763, D28917(3a), D49374(3b), Y12083 and AY858526(6a), D84262(6b), D84263(6d), D84264(6k).

### Statistical analysis

Results are expressed as means ± standard deviations or as percentages. Means between groups were compared by using the *t* test or the Student test. The frequency distributions of the different genotypes within groups were analyzed by the extended Mantel–Haenszel Chi square test. Associations between categorical variables were measured using Chi Square and Fisher’s Exact tests. Statistical analysis was performed using SPSS17.0 software (SPSS Inc., USA) and P values of 0.05 or less were considered significant.

## Results

### Demographic characteristics

**Fig. 1:**
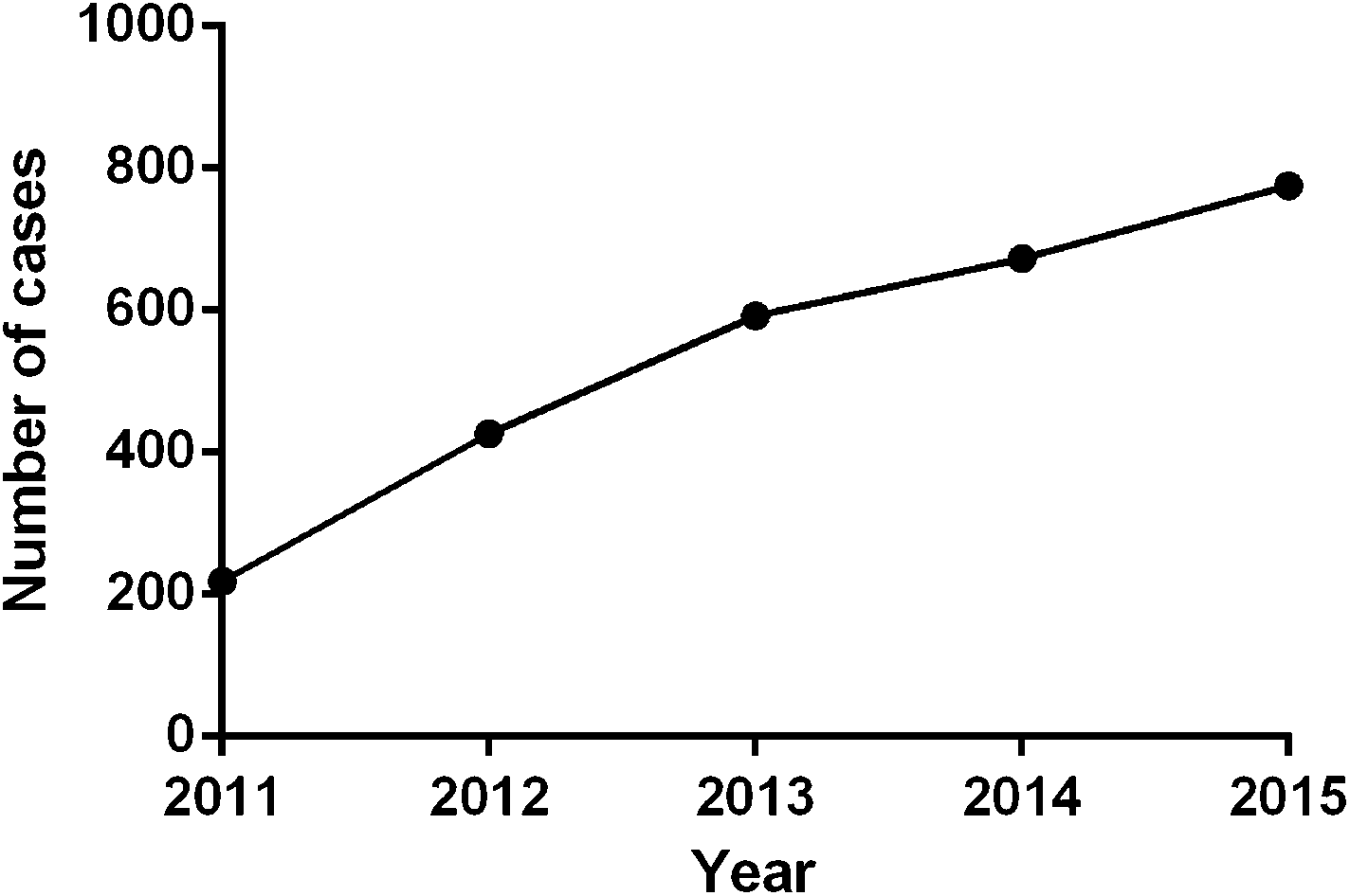
The prevalence in WuHan from 2011–2015

**Fig. 2:**
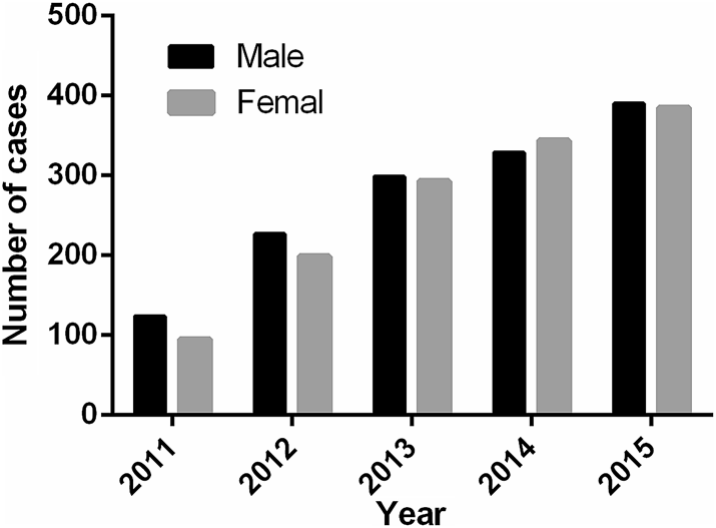
The prevalence in different gender in WuHan from 2011–2015

The main demographic characteristics of the patients are shown in Table 1. Of the 243,922 participants from 2011 to 2015 in WuHan city of China, 2685 cases were infected with HCV. Therefore, the sample size represents 1.1 % of the total number of HCV positive patients in WuHan during the study period. The number of HCV infection showed an increase with year, but the annual infection rate has remained similar. (χ^2^ = 2.94, *P* = 0.568; Fig. 1). Out of 2685 HCV-infected individuals of physical examination, 50.99 % (1369/2685) were males and 49.01 % (1316/2685) were females. The prevalence of males HCV infection significantly higher than that of females in 2011 (male 56.62 % vs female 43.38 %, RR = 1.31) and 2012 year (male 53.29 % vs female 46.71 %, RR = 1.14), respectively (Fig. 2). Routes of infection were investigated by questionnaire survey among the 2685 HCV seropositivity cases. In the present study, seven kinds of routes of infection are collected, including blood transfusions (18.14 %), Surgery (8.94 %), Hemodialysis (5.88 %), blood donation (3.05 %), dental therapy (2.61 %), Piercing/tattoo (1.64 %) and drug addiction (0.93 %).

**Table 1:** Demographic characteristics of patients in 2011–2015

Factors	2011	2012	2013	2014	2015
Age (old)	51 ± 16	50 ± 18	53 ± 10	52 ± 15	51 ± 13
Gender, number (%)					
Male	124 (56.62)	227 (53.29)	299 (50.51)	329 (48.89)	390 (50.32)
Female	95 (43.38)	199 (46.71)	293 (49.49)	344 (51.11)	385 (49.68)
Route of transmission, number (%)					
Unknown	128 (58.45)	253 (59.20)	356 (60.14)	409 (60.77)	433 (55.87)
Blood transfusion	43 (19.63)	75 (16.87)	97 (16.39)	116 (17.24)	156 (20.13)
Surgery	18 (8.22)	40 (9.20)	53 (8.95)	53 (7.88)	76 (9.81)
Dental therapy	5 (2.28)	11 (3.37)	16 (2.70)	18 (2.67)	20 (2.58)
Hemodialysis	11 (5.02)	27 (5.21)	34 (5.74)	35 (5.20)	51 (6.58)
Blood donation	7 (3.20)	13 (3.99)	20 (3.38)	23 (3.42)	19 (2.45)
Intravenous drug use	3 (1.37)	2 (0.61)	7 (1.18)	5 (0.74)	8 (1.03)
Piercing/tattoo	4 (1.83)	5 (1.53)	9 (1.52)	14 (2.08)	12 (1.55)

### Frequency of HCV infected patients according to age

**Fig. 3:**
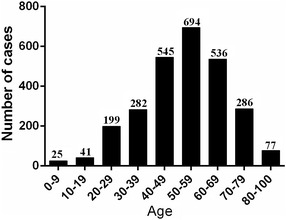
The prevalence in different ages in WuHan from 2011–2015

**Fig. 4:**
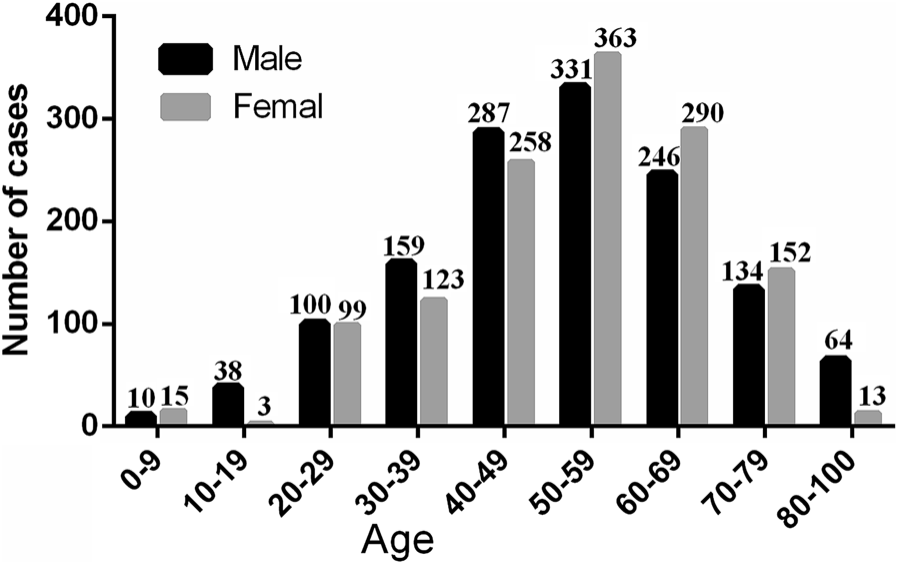
The prevalence in different age groups and gender

On basis of age, HCV infected patients were categorized into 9 age groups, as shown in Figs. 3 and 4. The prevalence of HCV infection showed different variations with age, as the highest incidence was shown for the age group 50–59 (25.85 % of 2685) and the lowest prevalence was 0–9 (0.93 % of 2685) (Fig. 3). The percentage of male positive samples were both higher in the age group 10–49 and 80–100 years, while most female positive samples were higher in the age group 0–9 and 50–79 years (χ^2^ = 25.52, *P* < 0.0001) (Fig. 4).

### Distribution of HCV genotypes in the study population

**Fig. 5:**
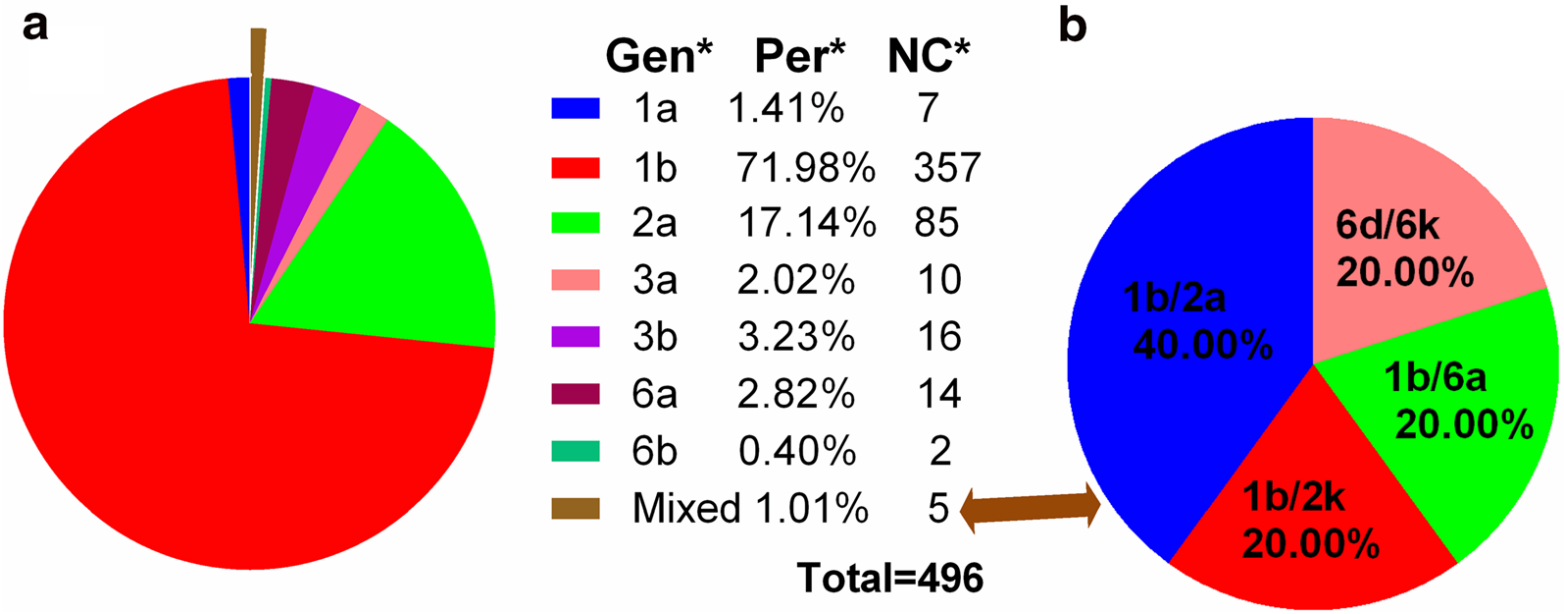
HCV genotype constituent ratio in WuHan; **a** shows HCV genotype constituent ratio in WuHan; **b** shows the constituent ratio of mixed genotypes

Figure 5 shows the frequency of HCV genotypes among the studied group. Four genotypes (1–3 and 6) and ten subtypes of HCV were identified in the studied population (Fig. 5). HCV genotype 1 was the most prevalent (73.39 %), followed by genotypes 2 (17.14 %), 3 (5.25 %), 6 (3.22 %). Genotype 4 and 5 was not detected in these patients. The most prevalent subtype was subtype 1b (71.98 %), followed by genotypes 2a (17.14 %) (Fig. 5a). Five patients (1.01 %) had mixed infection across the HCV subtypes: two cases within subtype 1b/2a, one within subtype 1b/2k, one within subtype 1b/6a and one case with subtype 6d/6k, which suggested that 1b mixed genotypes were more frequently observed (Fig. 5b).

### Frequency of HCV genotypes according to gender

**Fig. 6:**
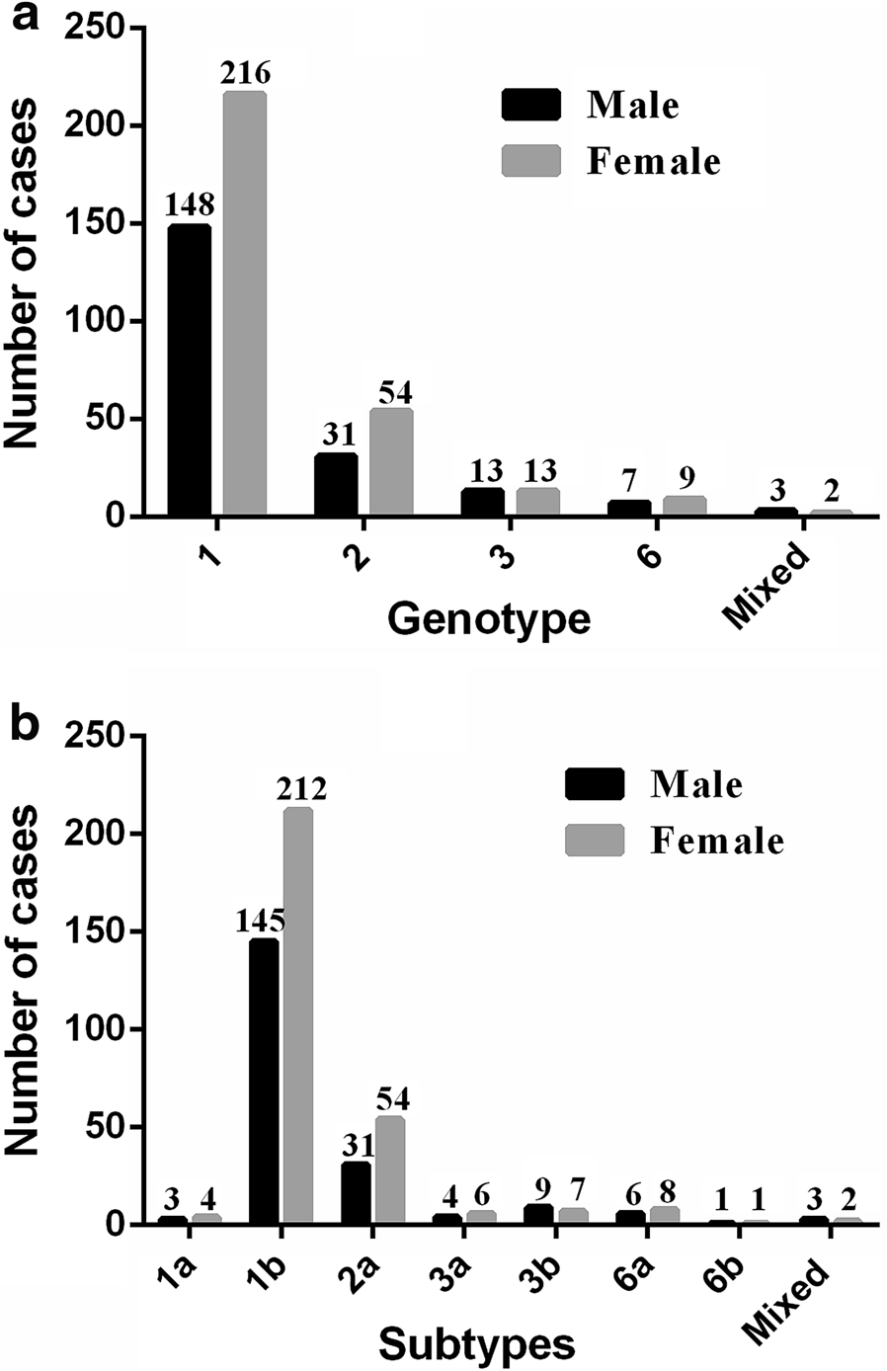
The HCV genotype and subtype distribution are related to the genders; **a** shows the HCV genotype distribution is related to the genders; **b** shows the HCV subtype distribution is related to the genders

Figure 6 shows the frequency of HCV genotypes between different genders groups. The frequency of all different HCV genotypes was higher in female patients compared to males (females/males: RR = 1.46), since the proportion of male was 40.7 % (202/496), while the female proportion was 59.3 % (294/496). Among all genotypes, genotype 1 was highest in both male (73.27 % of 202, genotype 1/the other genotypes: OR = 2.74) and female (73.47 % of 294, genotype 1/the other genotypes: OR = 2.77) patients, followed by genotype 2. Genotype 1 (male: 29.84 % of 496, vs female: 43.55 % of 496, χ^2^ = 20.07, *P* < 0.0001), genotype 2 (male: 6.25 % of 496, vs female: 10.89 % of 496, χ^2^ = 6.81, *P* = 0.009), and 6 (male: 1.41 % of 496, vs female: 1.81 % of 496, χ^2^ = 0.626, *P* = 0.401) were more common in female patients than males, while no significant gender differences were observed for genotype 6. In addition, the frequently of HCV genotype 3 was equivalent in male and female patients (male: 2.62 % of 26, vs female: 2.62 % of 26). Mixed genotypes were the least common genotype in both male and female patients (Fig. 6a).

Among the sub-genotypes, Subtype 1b was more frequent in both male (71.78 % of 202) and female (72.11 % of 294) patients, followed by 2a. Genotype 1b was more frequently observed in females than in males (male: 29.23 % of 496, vs female: 42.74 % of 496, χ^2^ = 19.64, *P* < 0.0001). Genotype 2a was more frequently observed in females than in males (male: 6.25 % of 496, vs female: 10.89 % of 496, χ^2^ = 6.81, *P* = 0.009). Other less frequent subtypes were 3b (1.81 %), 6a (1.21 %), 3a (0.81 %), 1a (0.60 %), 6b (0.20 %) and mixed (0.6 %) in male and 6a (1.61 %), 3b (1.41 %), 3a (1.21 %), 1a (0.81 %), 6b (0.20 %) and mixed (0.4 %)in females, which was no significant gender differences among them (*P* > 0.05) (Fig. 6b).

### Frequency of HCV genotypes according to age

**Fig. 7:**
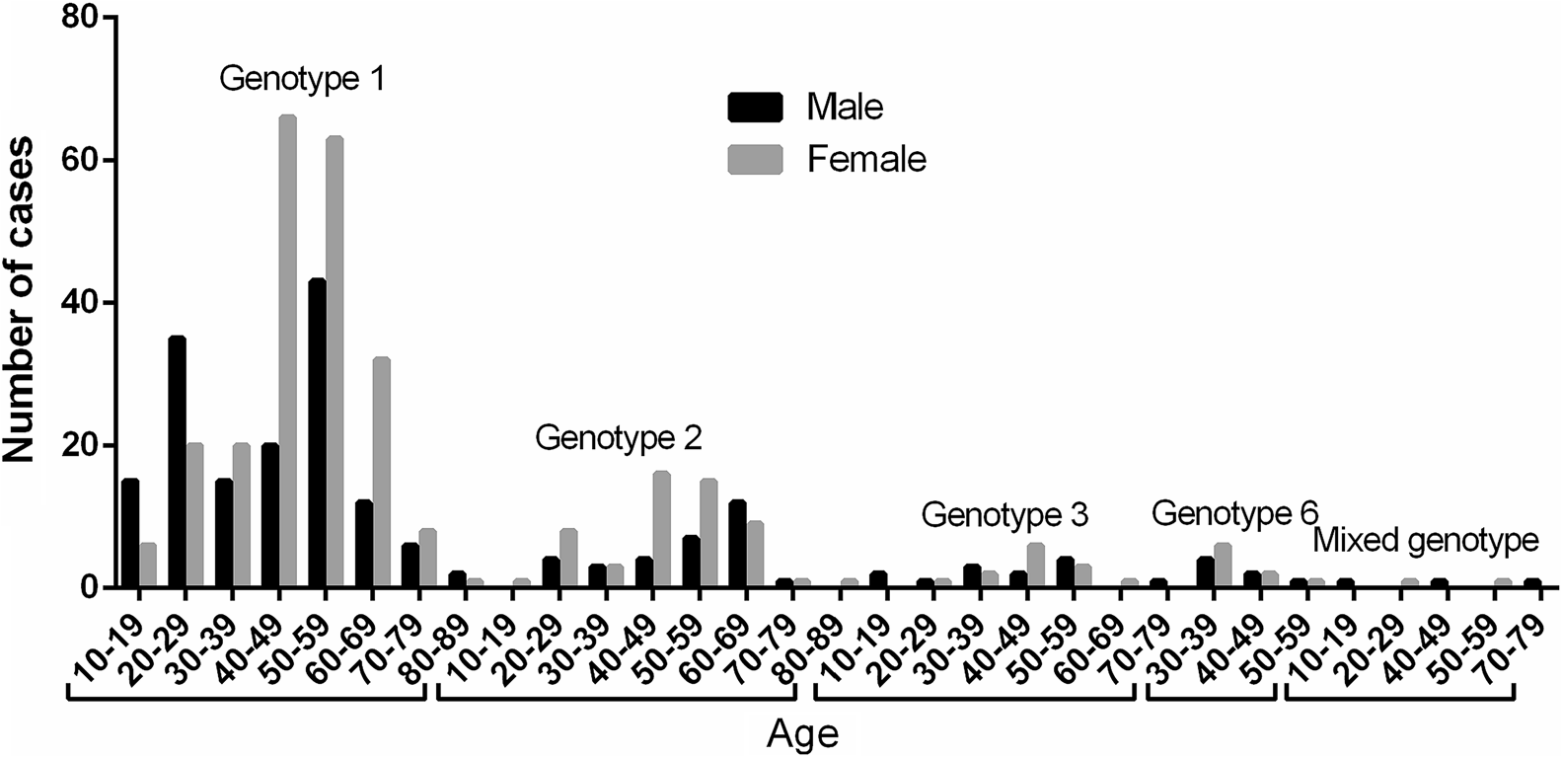
Frequency of HCV genotypes in different age groups

In order to compare the prevalence of HCV genotypes according to age in 2011–2015 year, the patients were categorized into 8 age groups, as shown in Fig. 7. Genotype 1 was most common in male patients of the age group 50–59 years (29.05 % of 148) followed in 20–29 years (23.65 % of 148), genotype 2 in the age group 60–69 (38.71 % of 31) and genotype 3 in the age group 50–59 (30.77 % of 13) and genotype 6 was most frequent in the age group 30–39 (57.14 % of 7), while Genotype 1 was most common in female patients of the age group 40–49 years (66/216, 30.56 %) followed in 50–59 years (63/216, 29.17 %), genotype 2 in the age group 40–49 (16/54, 29.63 %) followed in 50–59 years (15/54, 27.78 %) and genotype 3 in the age group 40–49 (6/13, 46.15 %), while genotype 6 was most frequent in the age group 30–39 (66.67 %). Three mixed genotype including 1b/2a, 1b/2k, 6d/6k at the age group of 10–19, 40–49 and 70–79 in male, while two mixed genotype including 1b/2a and 1b/6a were observed at the age group of 20–29 and 50–59 in female (Fig. 7).

### Frequency of HCV subtypes according to age and gender

**Fig. 8:**
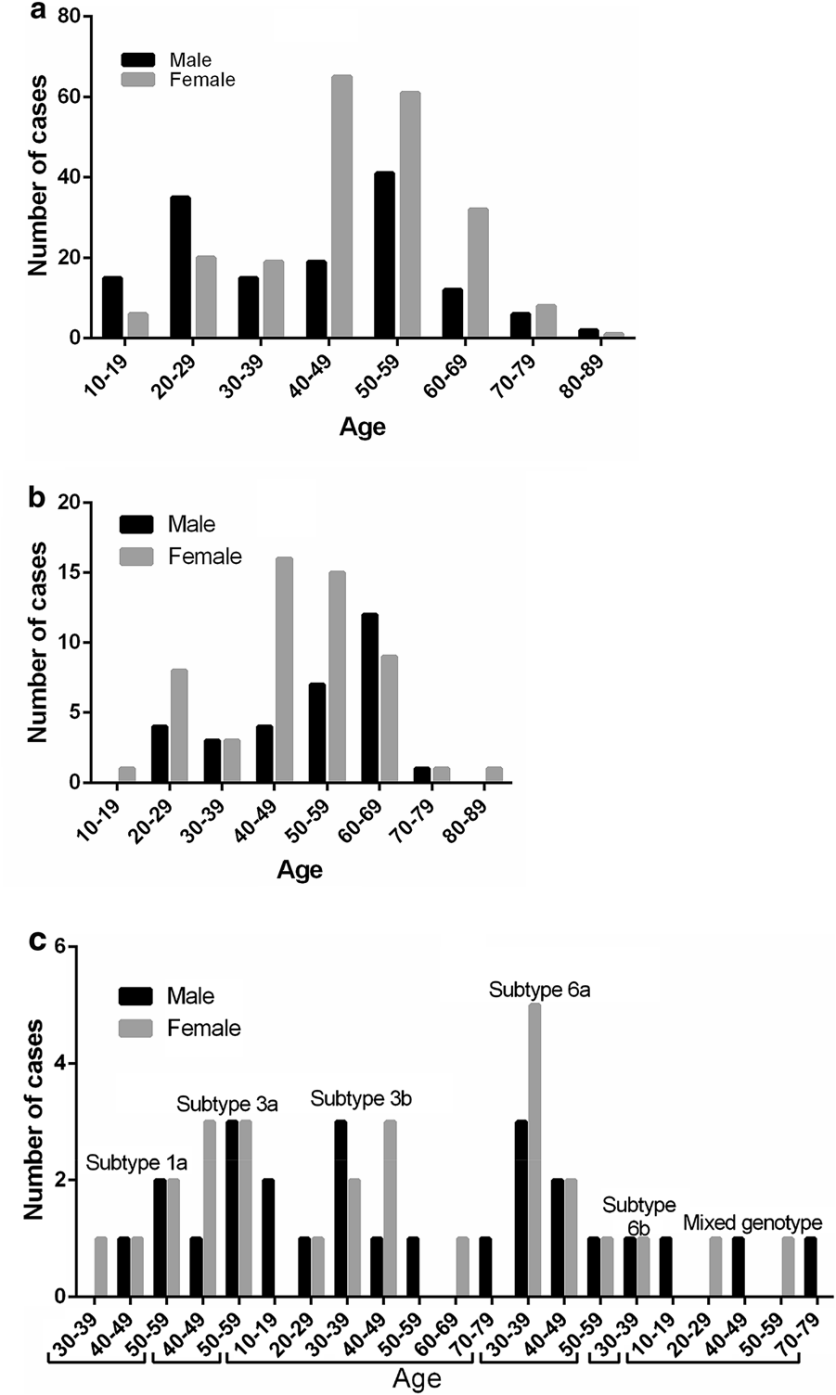
The distribution of HCV genotyping in different age and genders groups; **a** the results of HCV 1b genotyping in different age and genders groups; **b** the results of HCV 2a genotyping in different age and genders groups; **c** the results of HCV other genotyping in different age and genders groups

Subtypes 1b was most frequent in male patients of the age groups 50–59 (28.28 % of 145) and in female patients of the age groups 40–49 (30.66 % of 212). More importantly, genotype 1b was more frequently observed in younger (less than 29 years) male patients than female, while less frequently in older (more than 30 years old) male patients than female except the patients of the age groups 80–89 because of less number of cases, only 3 patients (χ^2^ = 26.72, *P* < 0.0001, Fig. 8a).

Subtypes 2a was more common in female patients who were 10–59 years old than males except the patients of the age groups 30–39 with similar between both, while less frequently in male patients over 60 years old except the patients of the age groups 70–79 with similar between both (χ^2^ = 5.13, *P* = 0.023, Fig. 8b).

Genotype 1a was only observed in female patients of the age groups 30–39, while it was similar in male and female patients of the age groups 40–49 and 50–59. Genotype 3a was more frequently observed in female patients than males of the age groups 40–49, and no difference was observed in 50–59. Genotype 3b was not observed in female patients of the age groups 10–19, 50–59 and 70–79 and in male patients of the age groups 60–69. Genotype 3b was more frequently in male patients than males of the age groups 30–39 than males, but less frequently in male patients than males of the age groups 40–49. At last, Genotype 3b was no difference between male and female patients of the age groups 20–29. Genotype 6a was more frequently observed in female patients than males of the age groups 30–39, while it was similar in male and female patients of the age groups 40–49 and 50–59. Genotype 6b was only observed in female and male patients of the age groups 30–39, but there was no difference between both. The mixed subtypes were only observed in male patients of the age groups 10–19, 40–49 and 70–79, and in female patients of the age groups 20–29 and 50–59 (Fig. 8c).

## Discussion

Due to lack of vaccine and effective therapy, limiting transmission is a primary strategy for the prevention and control of HCV epidemics. Since 1998, the blood donation law was legislated and the law requiring mandatory screening for anti-HCV before blood donation, improved control of blood transfusion in China has decreased HCV transmission (Chen et al. 2013; Gao et al. 2011). In this study, the number of HCV infection cases was increased, but the proportion of infections remained stable by year. The overall prevalence of HCV in WuHan during the study period was 1.1 %, which was lower than the national average infection rate. We also found that the overall percentage was no significant difference between men and women over the last 5 years, but HCV is more prevalent among females than males in 2011 and 2012 years. Among the 2685 cases, blood transfusions was a statistically significant risk factor associated with HCV infection in our study, similar to that observed in other countries such as the U.S. (Gower et al. 2014; W. H. Organization 2016).

Considering the age group distribution, the prevalence of HCV infection showed an gradually increased before 60 years old. The prevalence of HCV infection showed a decrease with age after 60 years old, which may due to the decline in physical mobility and economic strength that leads to decrease the number of elderly examination.

Genotypes play a crucial rule in assessing therapeutic decisions and approaches. As it has shown that the severity, prognosis of disease and response to therapy may vary according to the genotypes (Antaki et al. 2013; Wyles and Gutierrez 2014). For example, genotypes 1a and 1b tended to have more severe liver disease and lower response to interferon therapy (Wyles and Gutierrez 2014). More importantly, patients with genotype 1b are at higher risks for hepatocellular carcinoma (Al-Kubaisy et al. 2014). The present study was the first investigation of the frequency of HCV genotypes of different ages and genders in WuHan, which may contribute positively to refinement of HCV prevention and therapeutic programs. It was found that genotype 1 was identified as the predominant genotype affecting 71.98 % of the patients, followed by genotypes 2 (17.14 %), 3 (5.25 %), 6 (3.22 %). Genotype 4 and 5 was not detected in these patients. According to our present knowledge, HCV genotype 4 is widespread in the Middle East and Central Africa. Genotype 4 has been linked with increased incidence of cirrhosis and poor response to interferon therapy (Messina et al. 2015; Lu et al. 2005; Nguyen et al. 2010). Previous studies had showed the presence of 5a but rare status of this genotype (Afridi et al. 2014; Ahmad et al. 2010; Attaullah et al. 2011). Two study have reported that the most frequent genotypes were 3a (40.96 %), followed by 3b, and 1a in Pakistan and genotype 4 was the most common genotype, followed by genotype 1b, and 1a in Saudi Arabia, which is not consistent with our results (Al Ashgar et al. 2013; Idrees et al. 2009). However, the genotype 1 was consistent with previous studies of the HCV subtype distribution in China, Jordan (73.3 %), Iran (56.2 %), Turkey (87 %) and Israel (> 70 %) (Bdour 2002; Bokharaei-Salim et al. 2013; Bozdayi et al. 2004; Jahanbakhsh Sefidi et al. 2013; Shemer-Avni et al. 1998). In addition, mixed HCV genotype was observed in 1.01 % of the study population, which is common among patients who received multiple blood transfusions like patients with thalassemia and Sickle cell disease, which is a common disease in Pakistan (Idrees and Riazuddin 2008). A recent study indicated that there was no viral recombination events or mixed HCV infections in Chengdu, the capital of Sichuan Province in china. To the mixed genotype, 1b mixed genotype including 1b/2a, 1b/2k and 1b/6a, and 6d mixed genotype such as 6d/6k were found in our study. This may result in poor prognosis and poor response to antiviral therapy or relapse following antiviral treatment. In summary, the HCV sequences of HCV infected patients from WuHan were classified into ten subtypes, with the most frequent being subtype 1b (71.98 %), followed by 2a, 3b, 6a, 3a,1a, 6b and mixed genotype 1b/2a, 1b/2k, 1b/6a, 6d/6k. This is one of the few studies that have examined the distribution change of subtype 6a in China. Subtype 6a is largely responsible for the regional epidemic in Guangdong Province, which was estimated to have been transmitted via the IVDU route from Vietnam to the Chinese provinces of Guangxi and then to Guangdong (Larkin et al. 2007). Our study also showed the subtype-6a-infected population expanded from IVDU to the general population.

Previous studies from different countries have analyzed genotype distribution in relation to the genders. In Libya, the prevalence of HCV genotype 1 was found to be significantly associated with males, while genotype 4 has frequently been found in females (Brahim et al. 2012). Rouabhia et al. (2013) clearly show that there is no variation among HCV genotypes and gender as the different HCV genotypes were distributed with the same ratio in males and females. However, the frequency of various HCV genotypes was more prevalent among males than females in our study. For instance, the prevalence of HCV genotype 1 and 2 were found to be significantly associated with females, while genotype 6 has frequently been found in males. Moreover, the genotype 3 distribution is similar between male and female. For patients infected with mixed genotypes, statistical analysis was not performed due to the small number of patients in both groups. The present study also identified an increasing frequency of subtype 1b and 2a in female patients compared to males. Similarly, other subtypes were not performed due to the small number of patients in both groups.

The distribution of HCV genotypes may vary due to the age of the population. In some studies, it was determined that Subtypes 1a and 1b were more common among older patients (51–60 years old), whereas subtype 3b was the most common subtype among younger individuals (10 - 20 years old). Similar results were found by several recent studies, which indicate an increase in the frequency of HCV genotype 3 among younger population of Iran, Germany, Serbia and Montenegro and Slovenia (Jahanbakhsh Sefidi et al. 2013; Ross et al. 2000; Seme et al. 2009; Svirtlih et al. 2007). In the United States and western European countries, HCV non-genotype 1 is increasingly prevalent in younger patients and this is attributed to risk exposure differences (Cornberg et al. 2011). The results of our study showed that distribution of HCV genotypes varies with age. For instance, genotype 1 was more common in male between ages 10 and 29, while it was more prevalent in female between ages 30 and 79. The subtype 1b distribution between male and female is similar to genotype 1. Genotype 2 and subtype 2a were more common in female than male patients before 59 and over 80 years old, while more prevalent in male between ages 60 and 69, and has the equal proportions among 70–79 years old. To our knowledge, it is the first that evaluates the distribution of genotypes of HCV in WuHan, but it has some limitations, such as subtype with a relatively small sample size and a selection bias is possible given the use of the data from a single laboratory, but our study may be helpful for understanding the genotype distribution of HCV in WuHan and can provide important information about HCV prevalence among different ages and sexes.

In conclusion, we found that HCV 1b is a predominant genotype in WuHan and HCV prevalence and genotype distribution has a certain relationship with different age and gender. Further studies are needed to collect a large number of samples to estimate the different epidemiology of the HCV genotypes, because the sample size of non-genotype 1b and 2a is not large enough and other factors like disease history/monthly income/etc. are not included in our study. This study provides important information for the development of improved HCV prevention and control strategies.
